# A comprehensive analysis of antigen-specific autoimmune liver disease related autoantibodies in patients with multiple sclerosis

**DOI:** 10.1186/s13317-020-00130-4

**Published:** 2020-04-10

**Authors:** Zisis Tsouris, Christos Liaskos, Efthymios Dardiotis, Thomas Scheper, Vana Tsimourtou, Wolfgang Meyer, George Hadjigeorgiou, Dimitrios P. Bogdanos

**Affiliations:** 1 Department of Neurology, University General Hospital of Larissa, Faculty of Medicine, School of Health Sciences, University of Thessaly, Larissa, Greece; 2 Department of Rheumatology and Clinical Immunology, University General Hospital of Larissa, Faculty of Medicine, School of Health Sciences, University of Thessaly, Biopolis, Larissa 40500 Greece; 3 Institute of Experimental Immunology, Affiliated to EUROIMMUN AG, Lubeck, Germany; 4 Department of Neurology, University of Nicosia, Nicosia, Cyprus

**Keywords:** ANA, Autoantibody, Autoimmunity, Autoimmune hepatitis, Autoimmune rheumatic disease, Drug-induced liver injury, Liver diseases

## Abstract

**Introduction:**

Abnormal liver function tests are frequently seen in patients with multiple sclerosis (MS) and their origin at times is attributed to the possible co-occurrence or the de novo induction of autoimmune liver diseases (AILD), namely autoimmune hepatitis (AIH) and primary biliary cholangitis (PBC), but comprehensive analysis of AILD-related autoantibody has not been carried out.

**Aim:**

To assess the presence of AILD-related autoantibodies in a well-defined cohort of MS patients, and to assess their clinical significance.

**Materials and methods:**

133 MS (93 female) patients (102 RRMS, 27 SPMS, and 5 PPMS), mean age 42.7 ± 11.9 SD years, mean duration of disease 11.2 ± 7.2 years were studied. 150 age and sex-matched healthy individuals were tested as normal controls (NCs).Autoantibody testing was performed by indirect immunofluorescence (IF) using triple tissue and HEp-2, a multiparametric line immunoassay detecting anti-LKM1(anti-CYP2D6), anti-LC1(anti-FTCD), soluble liver antigen/liver-pancreas(anti-SLA/LP), AMA-M2, and AMA-MIT3 (BPO), PBC-specific ANA (anti-gp210, anti-sp100 and anti-PML), and ELISA for anti-F-actin SMA and anti-dsDNA antibodies.

**Results:**

Reactivity to at least one autoantibody was more frequent in MS patients compared to NCs (30/133, 22.6% vs 12/150, 8%) NCs (p = 0.00058). SMAs by IIF were more frequent in MS patients (18/133, 13.53%) compared to NCs (6/150, 4%, p = 0.002%). The AIH-1 related anti-F-actin SMA by ELISA were present in 21 (15.8%), at relatively low titres (all but three of the SMA-VG pattern by IF); anti-dsDNA in 3 (2.3%), and anti-SLA/LP in none; AIH-2 anti-LKM1 autoantibodies in 1 (0.8%, negative by IF), and anti-LC1 in none; PBC-specific AMA-M2 in 2 (1.5%, both negative for AMA-MIT3 and AMA by IF) and PBC-specific ANA anti-PML in 6 (4.5%), anti-sp100 in 1 (0.8%) and anti-gp210 in 1 (0.8%). Amongst the 30 MS patients with at least one autoantibody positivity, only 4 (3%) had overt AILD (2 AIH-1 and 2 PBC). Autoantibody positivity did not differ between naïve MS patients and patients under treatment.

**Conclusions:**

Despite the relatively frequent presence of liver autoantibodies, tested either by IF or molecular assays, overt AILD is rather infrequent discouraging autoantibody screening strategies of MS patients in the absence of clinical suspicion.

## Introduction

Multiple sclerosis (MS) is an autoimmune demyelinating disease, frequently characterized by concurrent autoimmune diseases, mainly including autoimmune thyroid disease, both Hashimoto’s thyroiditis and Graves’ disease, autoimmune rheumatic diseases, insulin-dependent diabetes mellitus, idiopathic Addison’s disease, atrophic gastritis, myasthenia gravis and inflammatory bowel disease [1–4]. It becomes apparent that in view of these con-current autoimmune diseases, several MS-unrelated autoantibodies have so far been reported.

Autoimmune liver diseases (AlLDs) appear less frequently, though reports suggest their prevalence being significantly higher to large populations of untreated MS compared to the general population [5–7]. Interferon-beta, used to treat MS of both the re- lapsing-remitting (RRMS) and of the secondary progressive form (SPMS), the two most common clinical phenotypes of MS, has been considered a usual trigger of autoantibodies, and at times of overt autoimmune disease [8]. In particular, sporadic cases of autoimmune hepatitis (AIH) have been described in untreated MS patients or following treatment with interferon-beta or other immunomodulatory therapeutic agents, including steroids and glatiramer acetate [9]. Despite the slightly increased prevalence of AIH, compared to that of the general population, routine screening for AILD of patients with established MS has been discouraged and is only recommended in those with abnormal liver enzymes or patients with a clinical suspicion of liver disease of unknown origin and drug-induced liver injury [5, 10].

It is not yet clear, whether AILD-related autoantibodies are de novo induced by MS-related immunomodulatory agents, pre-exist in the context of concurrent sub-clinical or clinical AILD or both [11–18].

Meticulous assessment of AILD-related autoantibodies [19, 20], paying attention to humoral responses against their molecular targets, in consecutive series of MS patients has not yet been performed.

The aim of the present study was to provide a complete profiling of these autoantibodies in patients from a single referral centre for MS patients in Central Greece, as this could initiate the impetus for assessing the diagnostic and clinical significance of AILD-related autoantibodies in this disease, providing novel insights as to whether routine autoab testing is needed for proper clinical decision making.

## Materials and methods

### Material

The study included 133 consecutive MS (93 female) patients (102 RRMS, 27 SPMS, and 4 PPMS—Primary Progressive MS), mean age 42.7 ± 11.9 SD years, mean duration of disease 11.2 ± 7.2 years. Table 1 shows the major clinical and laboratory findings of MS patients. Thirty patients (22.6%) were naïve (untreated) and 103 (77.4%) were on treatment including 35 patients (26.3% of the total) on interferon-β and 68 patients (51.1% of the total) on other immunomodulatory agents (24 patients with natalizumab; 20 with fingolimod; 16 with glatiramer; 5 with teriflunomide; and 3 patients with mitoxantrone).

**Table 1:** Major clinical and laboratory characteristics of patients with multiple sclerosis included to the study

	(n = 133)
Sex (M/F) (%)	40 (30.1%)/93 (69.9%)
Age (years) (mean ± SD)	42.7 ± 11.9
Age at diagnosis (years) (mean ± SD)	31.5 ± 10.4
(> 20/< 20 years)	132 (99.2%)/1(0.8%)
(> 30/< 30 years)	110 (82.7%)/23(17.3%)
(> 40/< 40 years)	72 (51.4%)/61(45.9%)
(> 50/< 50 years)	37 (27.8%)/96 (72.2%)
(> 60/< 60 years)	14 (10.5%)/119 (89.5%)
(> 70/< 70 years)	1 (0.8%)/132 (99.2%)
Type of MS (RRMS/SPMS/PPMS)(%)	102 (76.6%)/27 (20.3%)/4 (3%)
Duration (years) (mean ± SD)	11.2 ± 7.2
EDSS score	3.3 ± 2.1
Number of relapses	5 ± 3.6
Progression index	0.42 ± 0.56

EDSS: Expanded Disability Status Scale score, RRMS: Relapsing–remitting Multiple Sclerosis, SPMS: Secondary Progressive Multiple Sclerosis, PPMS: Primary Progressive Multiple Sclerosis

One hundred and fifty serum samples from healthy individuals tested as normal controls were also studied.

The study was been approved by the local Ethics Committee of the University General Hospital of Larissa, University of Thessaly (4/15-04-2016). Written informed consent was obtained by all participants.

## Methods

### Autoantibody testing

Autoantibody testing was performed by conventional indirect immunofluorescence (IIF) using triple liver kidney stomach tissue (cut off for positivity: 1/40) and HEp-2(cut off for positivity: 1/160). Reports for AILD-related autoantibodies by IIF included ANA of any pattern, SMA of any pattern (actin-SMA in liver tissue, vessels/glomeruli/tubuli in kidney tissue, AMA, anti-liver kidney microsomal (anti-LKM), and anti-liver cytosol (anti-LC).

A multiparametric line immunoassay detecting anti-LKM1(anti-CYP2D6), anti-LC1(anti-FTCD), soluble liver antigen/liver-pancreas(anti-SLA/LP), AMA-M2, and AMA-MIT3, PBC-specific ANA (anti-gp210, anti-sp100 and anti-PML) and anti-Ro52 was used as a molecular-based assay for the detection of AILD-related autoantibodies.

AIH-related SMA directed against F-actin were tested by an ELISA (Inova, San Diego, CA, USA), according to the manufacturer’s instructions [19]. All sera were tested at 1/101 dilution per manufacturers directives and according to our previously reported protocols [19].

Anti-ssDNA and anti-dsDNA antibodies testing was performed by ELISA, as per manufacturer’s instructions (Inova Diagnostics, San Diego, CA, USA), in accordance to the instructions of the manufacturer.

### Statistical analysis

All data are reported as percentages (%). Serum levels variation in each patients group was defined by mean and standard deviation (SD). Differences in categorical data between groups were tested by two-tailed Pearson’s Chi square and Fisher’s Exact Test after correction for continuity. Differences in numerical data between groups were tested by the two-tailed Student’s *t* test. *p*-values smaller than 0.05 were considered significant. All statistical calculations were performed with IBM SPSS Statistics 20 software.

## Results

Overall, 30/133 (22.6%) MS patients had at least one of the tested autoantibody specificities compared to 12 (8%) NCs (p = 0.00058).

ANAs by IIF were present in 8 (6.01%) patients with MS compared to 3 (2%) NCs (p = ns). The median titre of MS positive samples was 1/160 by IIF on HEp-2 (2 were positive at 1/80; 6 at 1/160 and 2 at a 1/320 dilution). Concerning IIF patterns, 4 had an homogenous pattern, 3 had fine speckled pattern and 1 one had multiple nuclear dots pattern (the same patient was also anti-sp100 positive, which is compatible with PBC-specific ANAs).

**Fig. 1:**
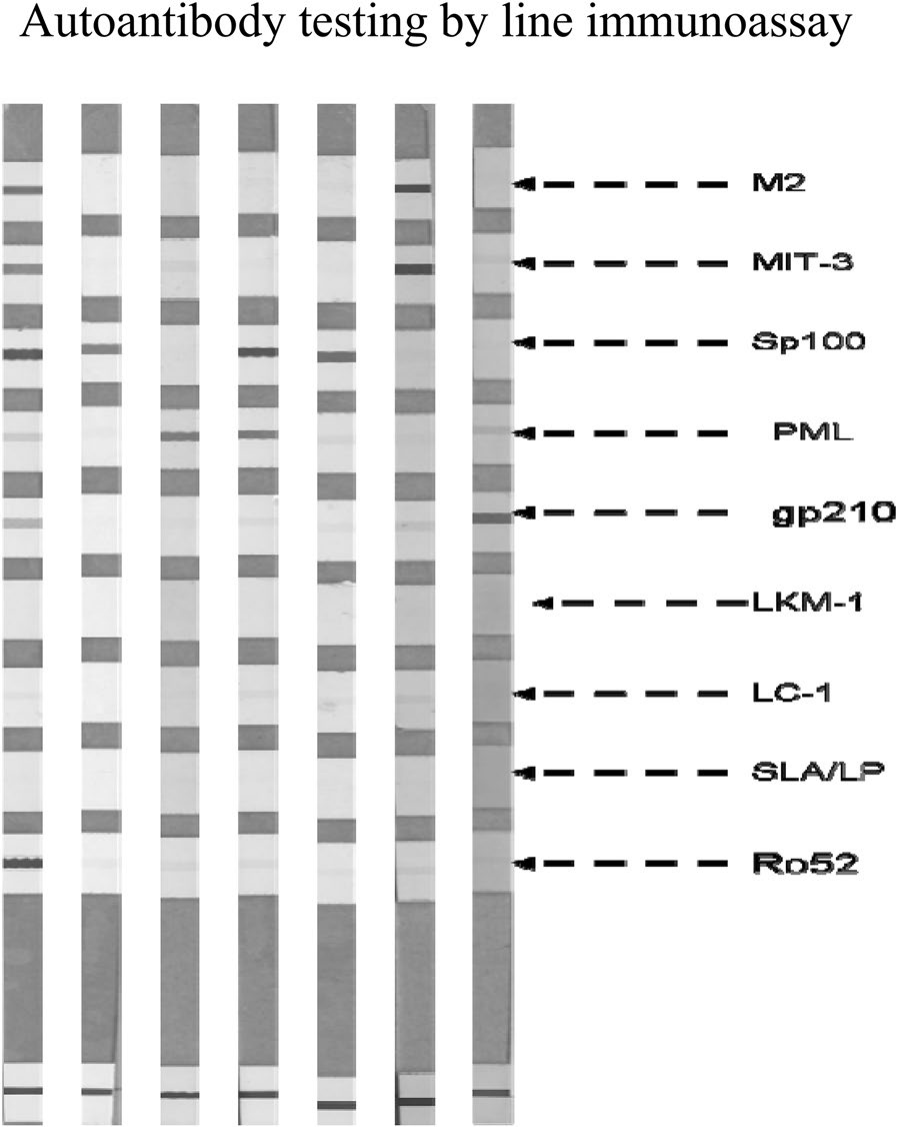
Representative cases of abs detection in patients with MS using a line immunoassay

SMAs by IIF were more frequent in MS patients (18/133, 13.53%) compared to NCs (6/150, 4%, p = 0.002%). Using the line liver blotting assay the following autoantibody’s reactivities were observed: AMA-MIT3 in 0 MS compared to 0 NCs (p = 1.00); AMA-M2 in 2 (1.5%) compared to 0 NCs (p = ns) (none of whom was positive for AMA-BPO by the same line immunoassay or by IIF); PBC-specific ANA anti-gp210 in 1 (0.8%) MS compared to 1 (0.7%) NCs (p = ns); PBC-specific ANA anti-sp100 in 1 (0.8%) MS compared to 0 (0%) NCs (p = ns); PBC-specific ANA anti-PML in 6 (4.5%) MS compared to 1 (0.7%) NCs (p = 0.053); anti-AIH2-specific anti-LKM1 (anti-CYP2D6) in 1% (0.8%, negative by IIF) MS compared to 0% NCs (p = ns); anti-LC1 (anti-FTCD) in 0% MS compared to 0% NCs (p = ns). AIH-specific anti-SLA/LP in 0% MS compared to 0% NCs (p = ns); and, anti-Ro52 in 2 (1.5%) MS compared to 2 (1.3%) NCs (p = ns). Representative cases of antibodies detection are showed in Fig. 1.

**Fig. 2:**
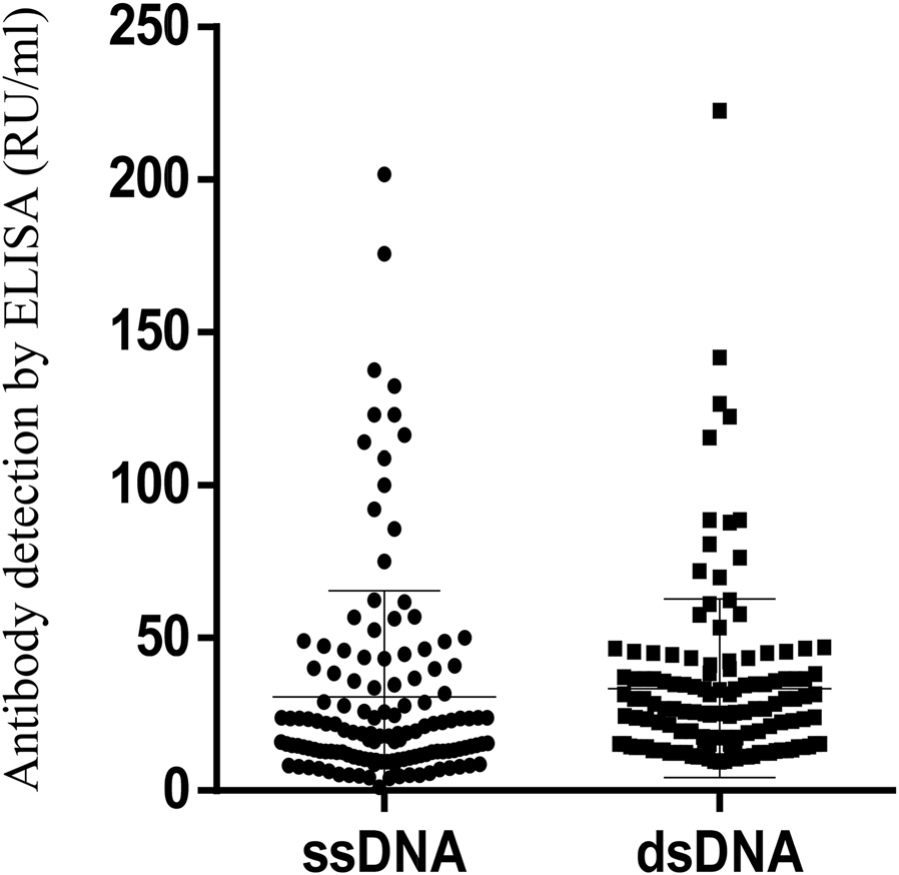
Levels of anti-ssDNA and anti-dsDNA and anti-F-actin abs in patients with multiple sclerosis

**Fig. 3:**
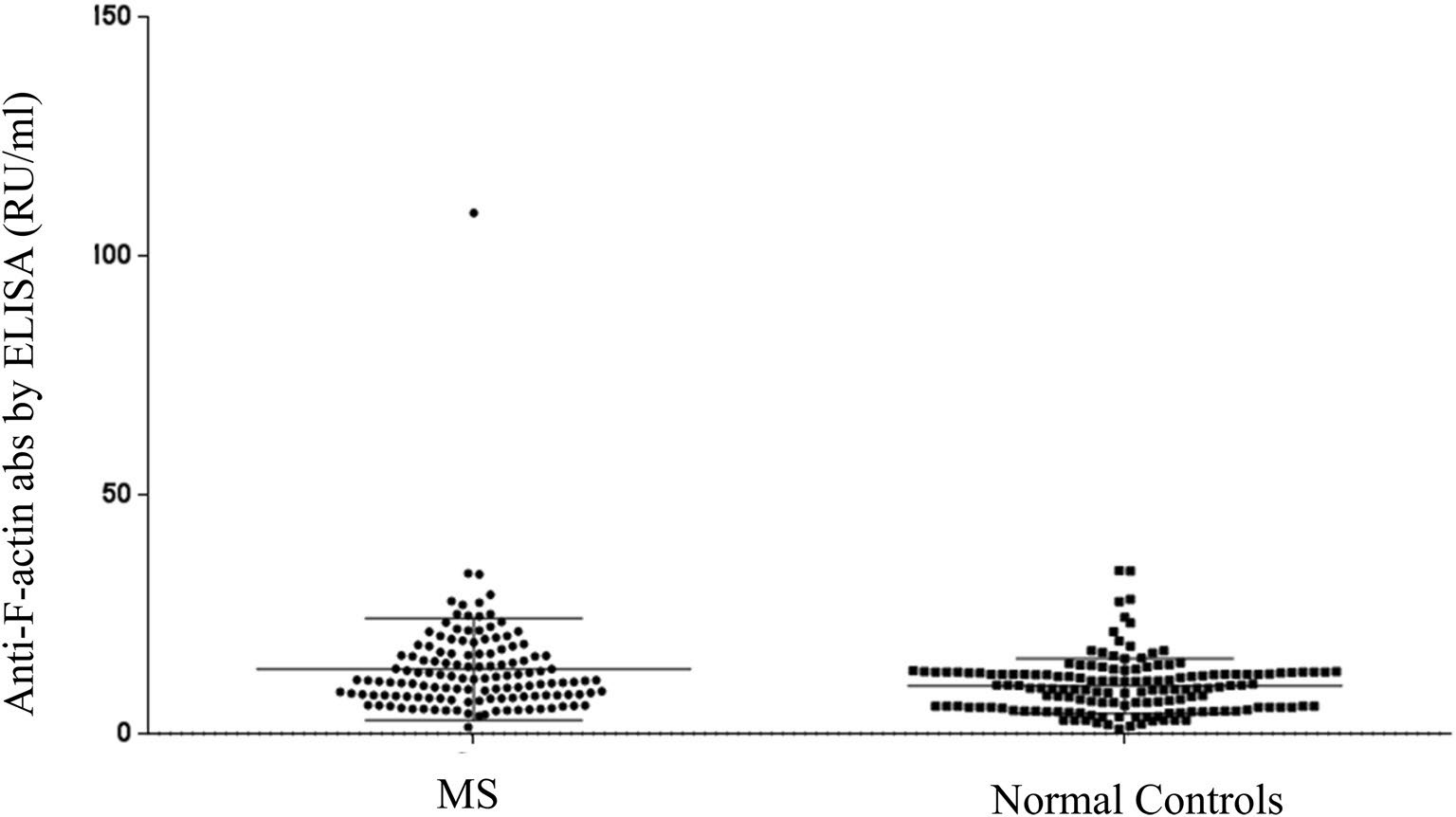
Levels of anti-F-actin antibodies in patients with multiple sclerosis (MS) and normal controls

By ELISA, AIH-1-specific anti-F-actin were more frequent in patients with MS compared to NCs (21/133, 15.8% vs 7/150, 4.7%, p = 0.0017) (all but three of them were tested positive for the SMA-VG pattern by IIF, amongst them 3 patients were also positive for SMA-F pattern by IIF); anti-ssDNA in 13 (9.77%) MS compared to 5 (3.33%) % NCs (p = ns); and anti-dsDNA in 3 (2.3%) MS patients compared to 2 (1.33%) NCs (p = ns), all three were also positive for anti-ssDNA. Levels of anti-ssDNA, anti-dsDNA and anti-Factin antibodies in patients with MS are showed in Fig. 2. The magnitudes of autoantibodies responses against F-actin did not differ between MS patients and NCs (13.53 ± 10.62 vs 11.81 ± 17.8 RU/ml p = ns, Fig. 3).

Of the 30 MS patients with at least one AILD-related autoantibody positivity only 2 (2/30, 6.7%) had overt AILD (1 with a known diagnosis of AIH-1 and 1 with a known diagnosis of PBC). One additional patient with detectable AMA and anti-gp210 antibodies in our cohort had also elevated liver enzymes and a diagnosis of PBC were confirmed. Eight of the 30 patients with MS and detectable AILD-related autoantibodies were followed up (median 73 months, range 39–126 months) and retesting of available serum samples was performed to witness the over time behavior of autoantibodies. Also, the biochemical and clinical profile of these patients that would potentially place a suspicion of a chronic liver disease, including AILD, was recorded. All patients who were tested had normal liver functions test during the period of follow up, with the exception of one female patient, tested positive for anti-F-actin, who showed transient mild elevation of transaminases, approximately 2 years after the date of experiment. Autoantibody positivity did not differ between naïve MS patients and patients under treatment: out of the 30 naïve MS patients, 8 (26.7%) had at least one autoantibody compared to 22/103 (21.4%, p = ns) MS under treatment. Regarding the most frequent autoantibody specificities, SMAs by IIF were present in 6/30 (20%) naïve MS patients compared to 12/103 (11.7%) under treatment (p = ns).

AIH-1-specific anti-F-actin (by ELISA) were present in 6/30 (20%) naïve MS compared to 15/103 (14.6%) MS under treatment (p = ns). No other association was found between autoantibody positivity and treatment status or type of MS-related treatment.

## Discussion

This is the first comprehensive study investigating the presence of AILD-related autoantibodies, both by IIF and antigen-specific assays (line blotting and ELISA) in a large cohort of Greek patients with MS. The major finding of our study is the peculiarly high prevalence of SMA, which rather unexpectedly targets F-actin, the predominant target of AIH-specific SMA with no apparent clinical or other laboratory evidence of underlying AIH. Except SMA, IIF testing reveals autoantibody positivity in a significant proportion of MS patients. However, testing of the seropositive cases using as antigenic substrate the well-defined molecular targets of these autoantibodies reveals only very few positive samples, raising questions as to whether these autoantibodies are of clinical relevance for the identification of suspected cases, who have or will develop AIH over time.

Various autoantibodies unrelated to the disease have been described in patients with MS, most of them are relevant to concomitant autoimmune diseases, such as thyroid autoantibodies. The presence of non-organ specific autoantibodies (NOSA) and in particular SMA and ANA have been reported extensively, at baseline and over time, in the latter case as result of immunomodulatory treatments which provoke autoantibody induction.

Schuller et al. [21] have detected DNA and RNA-specific autoantibodies, both in serum and CSF samples of MS patients. The notion that ANA can also be found in CSF has been challenged by more recent studies [22]. ANA in general, before or after treatment for MS has been reported in a range of 10-81% [2, 23, 24]. Seyfert et al. found ANA in 10.2% of MS patients, Heinzlef et al. in 30%, Aisen et al. in 35% and Dore-Duffy et al. in 81% of their MS patients [2, 23, 24]. The tremendous difference in positivity range relates to differences of autoantibodies cut-offs points used, sensitivity of the assays, techniques applied, patients cohorts biases (treatment status, older vs younger age cohorts), and other parameters comprehensively discussed over the years. Special attention has been given on whether IFN-b and other immunomodulatory agents can de novo induced NOSAs or whether the presence of ANAs in MS must alarm neurologists and place a clinical suspicion of autoimmune rheumatic disease highly likely. For example, the early study by Dore-Duffy et al. [14], which reported 81% ANA positivity considered an 1:8 dilution cut off for considering autoantibody positivity, 10 times lower than the 1:80 used by most other studies.

Barned et al. reported ANA in 27% of their RRMS cohort and have provocatively termed them “false-positive ANA”, as only rarely their presence confirms an underlying autoimmune rheumatic disease [25]. That term raised a heated debate as to whether “false positive” ANA can be an established/legitimate term [22–24]. Nonetheless, case studies reporting drug related (mainly due to IFN-b) autoimmune rheumatic diseases (and in particular SLE) led several authors to conclude that unmasking of underlying autoimmune rheumatic disease cannot be taken lightly over the course of MS [26–30]. The same point has been raised very recently for AIH and other AILDs diagnosed in patients with MS [9].

Several prospective studies have tackled this experimental question, at times testing large cohorts of patients before and after treatment for a significant period of time [15, 31, 32]. Durelli et al. have reported an 8.1% and 11%, prevalence of NOSAs, respectively before and after IFN-*b*1b [31]. Verdum et al. have conduced a multi-centre study assessing the dynamics of NOSAs over time in 156 MS patients treated with IFN-*b*1b (Betaferon^®^) [15].

While the prevalence of ANA and SMA at baseline was 4.7% and 1.3%, this increased to 5.8% and 3.7%, respectively. Baseline autoantibody positivity persisted at during treatment in the great majority of the cases, while those with de novo induction of autoantibodies demonstrated a continual increase over time to reach their maximum levels at 12 months after treatment [14–18, 22, 32–34].

The clinical significance of detectable ANA in relation to MS is highlighted in numerous studies failing to identify a clear association between their presence and disease activity, progression of disease or response to treatment [22]. SMA in serum specimens of MS patients has been known for long. Early and more recent studies have reported SMA in up to 50% of patients with MS [13], treated or not, remarking their relatively low/moderate levels, i.e. contrasting the SMAs of AIH-1 which can be as high as 1/10240 or higher [19, 35].

The novel finding we submit is that these low level SMAs in our cohort are of the SMA-VG (vessel glomeruli) pattern by IIF in kidney rodent tissue, a pattern which is also seen in AIH-1 and are directed against F-actin, the predominant target of AIH-1 specific SMA. We indeed have found by ELISA low titre anti-F-actin abs in 21 (15.8%), patients with MS (3 of them had also detectable SMA-F pattern by IIF), only 2 of whom had underlying AIH-1, the reference disease for F-actin SMA positivity. Of interest, local synthesis of SMA in the central nervous system has also been reported, but the mechanism for the induction remains unclear [36]. Intriguingly, myelin basic protein (MBP), a major MS-specific autoantigen, binds to negatively charged lipids on the cytosolic surface of oligodendrocyte membranes and can also polymerize actin, bundle F-actin filaments, and bind actin filaments to lipid bilayers through electrostatic interactions [37].

Whether MBP-bound F-actin serves as a neoantigen, responsible for the induction of anti-MBP abs but also F-actin SMA in patients with MS remains to be seen.

Finally, our study is probably the first to report on MS the presence of anti-Ro52 abs, an autoantibody marker of prototype autoimmune rheumatic diseases such as systemic lupus erythematosus and Sjögren’s syndrome. This autoantibodies, which is relatively frequent and can be found in non-autoimmune rheumatic diseases, as monospecificity (anti-Ro52 antibody positive in the absence of anti-Ro60) [38–42] as only present in 2 (1.5%) MS patients, a prevalence unexpectedly low, given the reported high prevalence of ANA in MS, autoimmune nature of the disease, and the relatively high prevalence of anti-Ro52 abs in the general population; all three points raising the logical expectation of a higher percentage rate for this autoantibody’s specificity in MS. None of these two patients had concomitant anti-Ro60 abs or clinical evidence of sicca syndrome.

Rather confusing was the evidence provided regarding other autoantigen specific reactivities, given that such autoantibodies are considered AILD-specific and are infrequently found in other irrelevant autoimmune disease, in the absence of current or future developed disease. Excluding 4 cases with known MS and concomitant AILDs, 7 had shown positivity PBC-associated autoantibodies such as anti-AMA BPO, PBC-specific ANA anti-gp210, anti-PML and one has shown AIH-2 anti-LKM1 (anti-CYP2D6) abs. The most frequent of those was the presence of anti-PML antibodies, which in PBC giving a characteristic multiple nuclear dot pattern [35, 43–45]. These autoantibodies were found in 6 of our MS patients, however, none of those had PBC or features compatible with cholestatic liver disease. Their titres were relatively low, significantly lower than those noted in patients with PBC and none of those had the specific indirect IF pattern, probably due to the fact that their magnitude was low. If the cut off was to be selected at 24 RU/ml instead of 11 RU/ml only 2 of those would still be considered positive. Selecting such a cut-off for anti-PML would decrease the sensitivity of the assay for anti-PML from 21 to 17%, as we published in a previous paper in patients with PBC (data not shown) [43]. In case these are no “false-positive” sera, we can only speculate that their presence can be considered as what it has been proposed as a generic a by-product of systemic immune dysregulation noted in MS, that could explain the presence of anti-PML and disease-irrelevant ANA [22]. Another tentative explanation would be that related to the ability of IFN-β to overexpress nuclear’s body PML, raising the possibility this to be a neoantigen exposed to immune system’s recognition in patients with MS [46]. If this overexpression becomes more evident under the influence of EBV infection, a known factor overexpressing nuclear’s body constituents such as PML remains to be seen [47]. Also, we cannot exclude that anti-PML antibody positivity in MS lacks biologically meaningful relevance, especially since it lacks an association with disease-specific indirect IF patterns of multiple nuclear dots.

In this category may also fall, the one case which has shown low-titre anti-LKM1 antibodies and did not raise any suspicion of AIH-2 or had virological evidence of hepatitis C virus infection (which at times has detectable anti-LKM1 abs).

In our cohort, 2 patients had history of confirmed co-occurrence autoimmune liver disease (one with autoimmune hepatitis and one with primary biliary cholangitis), while the diagnosis of PBC was confirmed in another one patients with abnormal profile of cholestasis. In the remaining cases, during the follow up period, no other diagnosis of AILDs were established.

Laboratorians but especially neurologists must be aware of these aspects, not only because rarely the presence of these autoantibodies (disease-specific or NOSAs) may indicate an underlying AILD but also because they may not hold any significant relevance and do not need to receive a careful follow-up in the absence of other clinical and laboratory features of AILDs.

## Data Availability

Material and data can be available upon request to the corresponding author.

## Abbreviations

Ab: Antibody
AIH: Autoimmune hepatitis
AiLD: Autoimmune liver diseases
AMA: Anti-mitochondrial antibody
ANA: Anti-nuclear antibody
AutoAb: Autoantibody
F-actin: Filamentous actin
IIF: Indirect immunofluorescence
SMA: Smooth muscle autoantibody
MS: Multiple sclerosis
PBC: Primary biliary cholangitis
PSC: Primary sclerosing cholangitis
